# Fractured Skull—Trephine in Case of an Insane Epileptic

**Published:** 1876-04

**Authors:** Thomas H. Kenan

**Affiliations:** 2d Assistant Physician, Georgia Lunatic Asylum


### FRACTURED SKULL—TREPHINE IN CASE OF AN INSANE EPILEPTIC.

By THOMAS H. KENAN, 2d Assistant Physician, Georgia Lunatic Asylum.

George Echols, colored; lunatic and epileptic; age 25; duration of epilepsy 4 years; convulsions usually occurred monthly, when he would have one or more very severely.

The cause is not known; has never had any severe injury by blows to head or otherwise; his general health is good. He was received here on the 17th of June, 1874, and put upon bromide of potassium and calcium treatment, and for the following few months his attacks were to some extent ameliorated.

On October 12th, 1874, he received a blow on the head from a brick, making a triangular wound in soft parts and crushing the skull in just the left of medium line in upper region of forehead, to the depth of three-quarters of an inch; diameter of fracture one and a-quarter inches. After carefully dissecting up the perostium and removing the particles of brick which were tightly impacked, Dr. Samuel G. White used his *very small* trephine, (manufactured by Tieman & Co. according to Dr. White's directions,) which allowed one lip of the forceps to enter and elevate the large plate which was tightly impacked. It required much care and trouble, owing to the fact that the plate was *much* larger on its inner than on its outer surface. With an ordinary instrument the loss of bone would have been great, but with the *very small* trephine, the operation was exceedingly gratifying. There were no symptoms of compression nor concussion, and but very slight hemorrhage. The parts were closed wtih silver suture; applied water dressing and solution of arnica; ordered an aperient, as bowels had not acted during the day; the next day bowels moved; he was cheerful; the third day the forehead was puffed during the morning, but that subsided by night; discontinued the use of solution of arnica; he was up and about in a few days, and with the exception of the escape of four small spiculae of bone, there was no hinderance of speedy recovery after the sutures were removed. He was discharged as restored February 9th, 1875, and employed as an attendant, where he has been up to this time (April 20th, 1875) doing duty, and has not had the first symptom of convulsion since he received the above named injury on October 12th, 1874. Having had them so frequently for four years prior to October, 1874, and never having received any blow on head or other serious injury prior to that time, makes us curious to know if the cessation of epilepsy is due to the violent injury sustained, or not.

